# Improvement of Kidney Function Following Unilateral Nephrectomy in a Patient With Cardiovascular-Kidney-Metabolic (CKM) Syndrome

**DOI:** 10.7759/cureus.83263

**Published:** 2025-04-30

**Authors:** Mrinal Asopa, Mohammad Kamgar, James Wilson, Sina Emami, Brian Shuch, Niloofar Nobakht

**Affiliations:** 1 Medicine, University of California Los Angeles, Los Angeles, USA

**Keywords:** albuminuria reduction, cardiovascular-kidney-metabolic (ckm), glucagon-like peptide-1 receptor agonist (glp-1 ra), sodium-glucose cotransporter-2 (sglt-2) inhibitors, unilateral nephrectomy

## Abstract

Cardiovascular-kidney-metabolic (CKM) syndrome is a significant contributor to the progressive decline in renal function, often leading to advanced chronic kidney disease. We report a 55-year-old man with a history of obesity, diabetes, and hypertension who was diagnosed with renal cell carcinoma (RCC) and underwent unilateral nephrectomy for his treatment, which led to decreased kidney function. The patient showed signs of improvement in glomerular filtration rate (GFR) and proteinuria after weight loss with combination therapy for CKM, including renin-angiotensin-aldosterone system (RAAS) inhibitors, sodium-glucose cotransporter-2 inhibitors (SGLT-2 inhibitors), and glucagon-like peptide-1 receptor agonists (GLP-1RA).

## Introduction

Cardiovascular-kidney-metabolic (CKM) syndrome is a complex medical disease containing a spectrum of interconnected factors, including hyperglycemia, insulin resistance, heightened activity of the renin-angiotensin-aldosterone system (RAAS), the generation of advanced glycation end-products, oxidative stress, and lipotoxicity [1]. It has affected more than 25% of US adults between 2015 and 2020 and was among the leading causes of death in 2021 [2].

Diabetes and obesity are significant contributors to the progressive decline in renal function, often leading to chronic kidney disease (CKD). Recent advances in pharmacotherapy for metabolic diseases have highlighted the potential of sodium-glucose cotransporter-2 inhibitors (SGLT-2 inhibitors) [3] and glucagon-like peptide-1 receptor agonists (GLP-1RA) [4] in improving patient outcomes. These therapeutic agents have demonstrated efficacy in managing hyperglycemia, managing obesity, and mitigating renal dysfunction, offering a promising approach for the comprehensive management of metabolic and renal conditions [3,4]. Further studies are warranted to explore their long-term impact on renal health and overall patient outcomes in cases with a history of decreased GFR after nephrectomy.

## Case presentation

A 55-year-old man with a past medical history of type II diabetes mellitus, obesity since age 50, as well as a history of renal cell carcinoma (RCC), for which a partial right nephrectomy was performed in 2019. He initially presented with body weight of 287 lbs, blood pressure (BP) 151/96 mmHg, urine albumin-to-creatinine ratio (UACR) of 2,279 mg/g, HbA1c of 7.6%, creatinine (Cr) of 2.43, blood urea nitrogen (BUN) of 34 and estimated glomerular filtration rate (eGFR) of 29 mL/min/1.73 m². GFR was seen to decrease due to comorbidities as well as due to partial nephrectomy.

Initially, the RAAS blocker was up-titrated (benazepril 20 mg to 40 mg). Two months later, a SGLT-2 inhibitor was added to his regimen with dapagliflozin 5 mg up-titrated to 10 mg for maximum antiproteinuric effect. Eight months later, GLP1-RA started (Tirzepatide 15 mg/0.5 mL) for weight loss. 15 months following the above management, the patient lost 40 lbs, his BP and HbA1c normalized, followed by an increase in eGFR, resulting in an improvement from CKD stage IV to stage IIIB and a decrease in proteinuria (Table 1).

**Table 1:** Metabolic parameters and kidney functions before and after triple therapy BMI - Body Mass Index, HbA1c - Hemoglobin A1C, eGFR - Estimated Glomerular Filtration Rate

Test	Reference range	Prior triple pharmacotherapy	After 15 months of combination therapy
Weight (Ibs)	-	287	247
BMI (kg/m^2^)	18.5-24.9	35	30
HbA1c (%)	<5.7%	7.6	5
Urine microalbumin/cr (mg/g)	<30	2279	893
eGFR (mL/min/1.73 m^2^)	>89	29	43
Blood pressure (mmHg)	<120/80	151/96	127/80

Of note, labs were checked in eight to 12-week intervals at the CKD clinic and showed an ongoing interval of improvement in microalbumin/creatinine ratio: 2,279 (H) > 1,889 (H)> 1,090 (H)>1,037 (H) > 893 (H) > 360 (H). This finding offers the possible utility of triple therapy of RAAS blocker, SGLT-2 inhibitor, and GLP1-RA for improving renal function in patients’ population with CKM and reduced eGFR due to unilateral nephrectomy with CKM.

## Discussion

SGLT-2 inhibitors and GLP-1RA have been gaining more attention for their renal benefits, particularly in managing diabetes, weight loss, and proteinuria in patients with CKM [3,5]. As noted in Table 1, our patient demonstrated significant improvement in renal indices along with improvement in metabolic markers. ​ This case also demonstrates improvement from CKD G4/A3 to stage G3B after initiation of SGLT-2 inhibitor and GLP-1RA, along with a combination of the maximum tolerated dose of RAAS blocker, which the patient was already on previously. This improvement is especially noteworthy considering the patient’s history of unilateral nephrectomy. SGLT-2 inhibitors have been approved for diabetic and nondiabetic patients at risk of CKD progression as well as for patients with heart failure. The specific mechanism includes increased distal renal NaCl delivery, causing an increased afferent tone, thereby reducing the intraglomerular pressure and glomerular hyperfiltration. The renal protective effects are thought to be due to inhibition of oxidative stress and inflammation as well as induction of natriuresis [3,5].

GLP-1RA has become a promising option in the use of CKD especially patients with diabetic kidney disease (DKD) [4]. The reno-protective effects are thought to be mediated by anti-inflammatory, antifibrotic mechanisms and hemodynamic impacts [6]. Recent finding suggests that GLP-1RA may reduce kidney disease progression in type 2 diabetes or overweight/obesity regardless of CKD status [7].

Findings suggest that the combination of SGLT-2 inhibitors and GLP-1RA may offer broader renal benefits in diabetic cases with obesity. Furthermore, there are other conditions, such as hypertensive nephrosclerosis, where GLP-1RA has shown improvement, but further studies are needed to fill the gap of knowledge for GFR improvements in this population with obesity and resistant hypertension [8].

The observed improvement in renal function highlights the potential of these agents to positively impact overall kidney health and contributes to improvement in renal function in unexplored patient populations, such as patients with chronic kidney disease, as well as a history of nephrectomy with decreased GFR after unilateral nephrectomy.

## Conclusions

Our case highlights a patient with CKM syndrome, including obesity, diabetes, hypertension, and CKD, with a history of unilateral nephrectomy due to RCC. The addition of an SGLT-2 inhibitor and a GLP-1RA to the maximum dose of a RAAS blocker led to improvements in metabolic markers and renal indices following weight loss. This approach is particularly significant as it is widely utilized across various healthcare settings, especially for patients with CKD and diabetes. We suggest further exploration of the benefits of triple therapy in this patient population with nephrectomy and reduced GFR, aiming to improve outcomes related to end-organ damage associated with CKM syndrome.
